# Impact of non-gated computed tomography on the timing of invasive strategy of patients with non-ST-elevation acute coronary syndrome

**DOI:** 10.3389/fcvm.2023.1266767

**Published:** 2023-11-20

**Authors:** Masatsugu Miyagawa, Riku Arai, Kurara Takahashi, Yuki Nakajima, Shohei Migita, Saki Mizobuchi, Yudai Tanaka, Katsunori Fukumoto, Tomoyuki Morikawa, Takashi Mineki, Keisuke Kojima, Nobuhiro Murata, Mitsumasa Sudo, Yasuo Okumura

**Affiliations:** Division of Cardiology, Department of Medicine, Nihon University School of Medicine, Tokyo, Japan

**Keywords:** coronary angiography, creatine kinase, non-gated computed tomography, non-ST-elevation acute coronary syndrome (NSTE-ACS), perfusion defect, non-ST-elevation myocardial infarction (NSTEMI)

## Abstract

**Background:**

This study aimed to examine the clinical role of non-gated computed tomography (CT) in ruling out fatal chest pain in patients with non-ST-elevation acute coronary syndrome (NSTE-ACS), with a focus on the time of arrival at the hospital to coronary angiography (CAG) and peak creatine kinase (CK) levels.

**Methods:**

We retrospectively examined 196 NSTE-ACS patients who were admitted with urgently diagnosed NSTE-ACS and underwent percutaneous coronary intervention between March 2019 and October 2022. The patients were divided into three groups, namely, non-CT group, CT and defect− group, and CT and defect+ group, based on whether they underwent a CT scan and the presence or absence of perfusion defects on the CT image.

**Results:**

After the initial admission for NSTE-ACS, 40 patients (20.4%) underwent non-gated CT prior to CAG. Among these 40 patients, 27 had a perfusion defect on the CT scan. The incidence of contrast-induced nephropathy was not different among the three groups. The CT and defect+ group had a shorter arrival-to-CAG time than that of the non-CT group. In NSTE-ACS patients with elevated CK levels, the CT and defect+ group had lower peak CK levels than those in the non-CT group.

**Conclusion:**

NSTE-ACS patients with perfusion defects on non-gated CT had a shorter time from arrival to CAG, which might be associated with a lower peak CK. Non-gated CT might be useful for early diagnosis and early revascularization in the clinical setting of NSTE-ACS.

## Introduction

Acute coronary syndromes (ACSs), which include ST-segment elevation myocardial infarction and non-ST-elevation ACS (NSTE-ACS), are caused by a sudden reduction in blood supply to the myocardium. In patients with NSTE-ACS, the timing of invasive coronary angiography (CAG) followed by percutaneous or surgical revascularization is crucial (1–3). Nonetheless, diagnosing NSTE-ACS at the initial presentation can be challenging because it may not always present with symptoms, typical electrocardiographic changes, or elevated cardiac enzymes. It also is important to rule out the other life-threatening causes of chest pain, such as due to aortic dissection, pulmonary embolism, or tension pneumothorax (1, 2, 4). Non-gated chest-enhanced computed tomography (CT) is a commonly used imaging modality for evaluating pulmonary artery or aortic disorders. The non-gated CT-derived myocardial perfusion defect has been shown to indicate the presence of acute myocardial infarction (5). In our institution, we reported the potential utility of the non-gated CT in the early diagnosis of NSTE-ACS by detecting a myocardial perfusion defect in 2019 (6, 7). Thus, clinicians at our institution often utilize non-gated CT for ACS evaluation. The clinical impact of non-gated CT, which is not general in ACS practice, is unknown. We, therefore, hypothesized that an early diagnosis of NSTE-ACS using non-gated CT during the initial examination could reduce myocardial damage by enabling early revascularization. The purpose of this study was to examine the clinical role of non-gated CT in ruling out thief-threatening chest pain in patients with NSTE-ACS.

## Materials and methods

### Study patients

We investigated 211 consecutive patients who were admitted urgently to Nihon University Itabashi Hospital, Tokyo, Japan, between March 2019 and October 2022 with suspected NSTE-ACS. These patients underwent CAG for a definitive diagnosis. All patients were diagnosed and treated by clinicians based on current guidelines (2, 3, 8). ST elevation was evaluated by synthesized 18-lead electrocardiography. As mentioned above, these clinicians are well-trained in detecting perfusion defects. Our institution is at any time available for emergency CAG. Heart failure, if present at the time of the initial presentation, was classified by severity according to the Killip classification (9).

**Figure 1:**
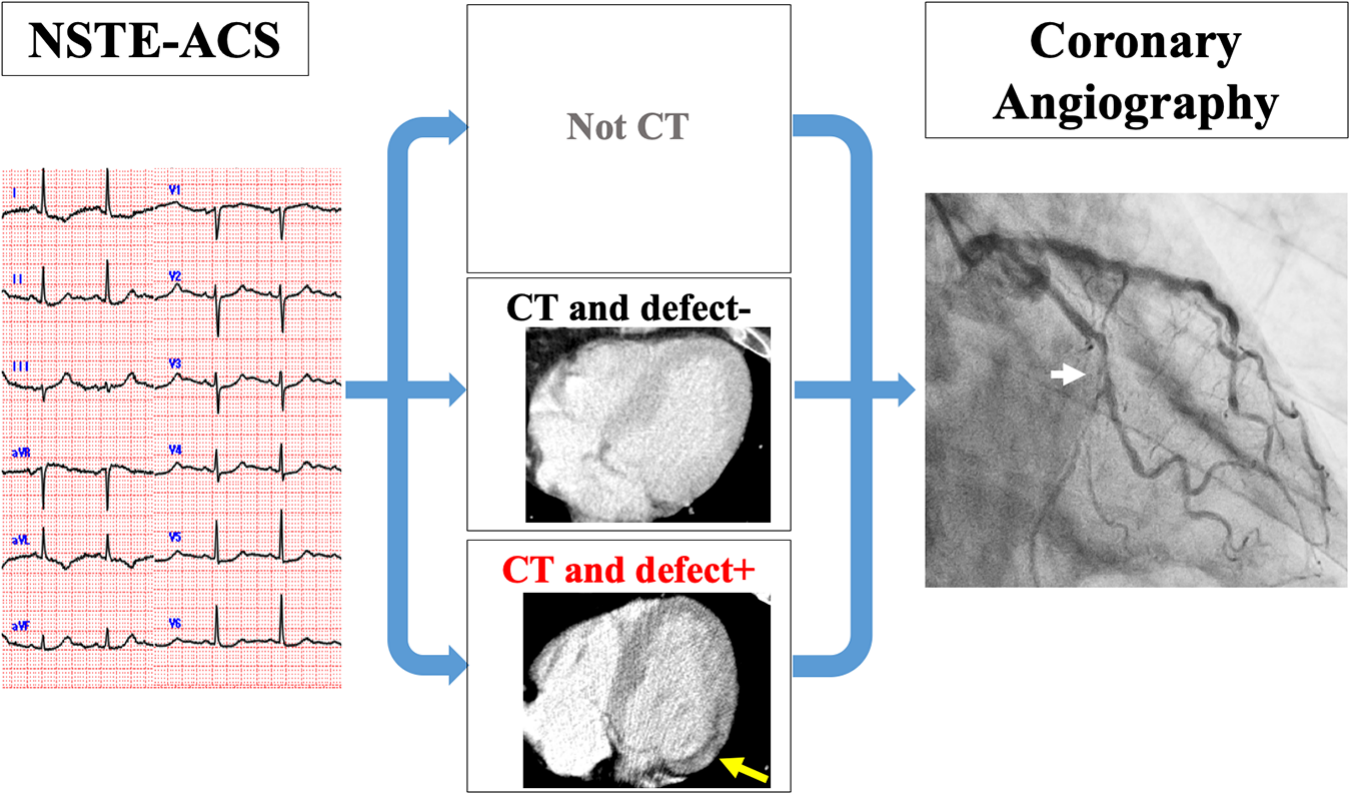
Clinical setting of the study. All patients were diagnosed with NSTE-ACS by CAG. They were divided into three groups: groups who did not undergo CT (non-CT), groups with CT and no perfusion defect on CT (CT and defect−), and groups with CT and perfusion defect on CT (CT and defect+). CT, computed tomography; NSTE-ACS, non-ST-elevation acute coronary syndrome; CAG, coronary angiography.

We excluded a total of 15 patients from the initial cohort of 211 patients. These exclusions consisted of six patients who underwent either coronary computed tomography angiography (CCTA) or myocardial single-photon emission CT immediately prior to CAG, three patients who were deferred for CAG due to critical bleeding that could not be managed with blood transfusions, one patient who was deferred for CAG due to acute kidney injury, one patient who underwent CAG and was scheduled for coronary artery bypass grafting, one patient who underwent percutaneous coronary intervention (PCI) that was changed to optimal medication due to technical difficulties, and three patients who required CAG but were unable to undergo the procedure at the appropriate timing due to the need for isolation related to COVID-19. As a result, a total of 196 patients were included in the final analysis of the study. These enrolled patients were then divided into three groups based on whether patients underwent a CT scan and the presence or absence of perfusion defects on the CT image (Figure 1). In our hospital, we have implemented a clinical system that involves conducting an early invasive strategy when a non-gated CT scan indicates a perfusion defect, referring to past reports (6).

The study protocol was approved by the Ethics Committee of Nihon University Itabashi Hospital (RK-2021121-1) and was in accordance with the ethical standards of the institutional research committee and the 1964 Declaration of Helsinki and its later amendments or comparable ethical standards. An opt-out system was used to obtain the consent of the patients for the use of their clinical data for research purposes.

### Non-gated chest CT

A total of 40 patients underwent non-gated CT prior to CAG after the initial admittance for NSTE-ACS. Contrast-enhanced CT was performed using a 64-section CT scanner (Brilliance CT 64-Channel scanner; Philips Medical Systems, Andover, MA, USA). An intravenous bolus of non-ionic contrast medium (Iopamiron; Bayer, Leverkusen, Germany) was injected through an arm vein. The dose of contrast medium was approximately 1.5 mL/kg (521 mg I/kg) of body weight. Four patients underwent CT with the general protocol as follows: the injection rate of the injector was calculated to inject the entire volume of contrast agent in 60 s, and the scanning delay was calculated by monitoring the main pulmonary artery. The scanning parameters were as follows: tube voltage, 120 kV; tube current, 200–400 mA depending on the size of the patient; gantry rotation time, 500 ms; the number of slices per rotation, 64; and individual detector width, 0.625 mm. The remaining 36 patients underwent CT with the aorta protocol as follows: the injection rate of the injector was calculated to inject the entire volume of contrast agent in 30 s, and the scanning delay was calculated by monitoring the main pulmonary artery. The scanning parameters were as follows: tube voltage, 100 kV; tube current, 200–400 mA depending on the size of the patient; gantry rotation time, 500 ms; number of slices per rotation, 64; and individual detector width, 0.625 mm. Beta-blockers were not used to prepare for CT. CT was performed under the above conditions and adjusted as needed.

### Image analysis

Two independent readers (MM and RA) blinded to patient clinical characteristics evaluated myocardial perfusion defects on non-gated CT, and another reader (KF) evaluated it in cases in which the previous readers had already known their clinical characteristics. Finally, a consensus was reached in cases of disagreement on the interpretation of perfusion defects. The assessment of perfusion defect was good reproducibility as indicated by Cohen's kappa (*κ*) of 0.79 (CI, 0.60–0.98) for the perfusion defect on non-gated CT.

### The time from arrival at the hospital to CAG

The time from arrival at the hospital to CAG was retrospectively obtained from the medical records of the patients. The start of CAG was defined as the time of entry into the angiography suite. CAG was performed using standard techniques with digital image acquisition and storage. Temporary pacing and mechanical circulatory support were all administered after entry into the angiography suite. Significant stenosis was defined as coronary stenosis >50%. The contrast agent used was iopamidol (Oypalomin 370, Konica Minolta, Tokyo, Japan) unless there was a reason not to use it.

### Laboratory studies

Normal upper limits of CK were defined as 248 IU/L and 153 IU/L for male and female patients, respectively. The CK levels were assessed on admission and at 4-h intervals for the first 24 h reaching the peak. Other cardiac enzymes were also used as a reference to accurately define CK peak out. A positive troponin I (cTnI) was defined as 0.010 ng/mL or greater. Contrast-induced nephropathy (CIN) was defined as an increase in serum creatinine of 0.5 mg/dL above baseline or serum creatinine ≥125% of baseline within 48 h of PCI (10). Patients with a history of maintenance hemodialysis were excluded from the incidence of CIN. Patients selected for urgent invasive treatment (i.e., within 2 h of arrival at CAG) were counted. We divided the value of serum creatinine and NT-proBNP in non-dialysis patients into two groups according to the cutoff value of over 1.5 mg/dL (10) and 900 pg/mL (11) for the risk of CIN and symptomatic heart failure, respectively, as generally reported.

### Evaluations and study outcomes

We evaluated the relationship between the presence of non-gated CT after hospital admission and the time of arrival at the hospital to CAG and the effects of CT and perfusion defects on peak CK in NSTE-ACS patients. Also, CT findings of perfusion defects and each type of NSTE-ACS as non-ST-elevation myocardial infarction with elevated CK (NSTEMI CK+), NSTEMI with only positive cTnI (NSTEMI CK−), and NSTE-ACS without elevated cTnI (NSTE-ACS UA) were evaluated. At least cTnI was measured on admission, immediately after PCI, and 12–24 h after PCI.

### Statistical analysis

Continuous variables were presented as the average value with the standard deviation or median with the interquartile range (IQR) as appropriate, and categorical variables were presented as the number and percentage of patients. To compare the three groups, we used the analysis of variance, the Kruskal–Wallis test, or the chi-square test, as appropriate. As a subgroup analysis, we compared the variables between the non-CT group and CT and defect+ group using the chi-square test, Fisher exact test, Student's *t*-test, or Wilcoxon rank sum test as appropriate. The univariate and multivariate logistic regression analysis was used to find the factors associated with CAG within 2 h of arrival and predictors of performing CT. In the multivariate analysis, we constructed models to adjust for the effects of clinically relevant factors including age, diabetes, tracheal intubation, creatinine ≥1.5 mg/dL, NT-proBNP ≥900 pg/mL, NSTEMI, and non-gated CT (8). In the multivariate analysis, the predictors of performing CT include age, NT-proBNP ≥900 pg/mL, creatinine ≥1.5 mg/dL, and left circumflex artery (LCX). Receiver-operating characteristic (ROC) curve analysis for perfusion defect on CT was performed, and the area under the ROC curve (AUC) was calculated. The optimal cutoffs were defined as the values of the ROC curves that were closest to the upper left corner. The effects of the variables were reported through the odds ratio (OR) with a 95% confidence interval (CI). A *P*-value of 0.05 was considered statistically significant. All analyses were performed with JMP Pro 16 software (SAS Institute, Cary, NC, USA).

## Results

### Patient characteristics

Based on CT and its finding of a perfusion defect, 196 eligible patients were classified into those who did not undergo CT (non-CT group, *n* = 156), those who underwent CT and did not have a perfusion defect (CT and defect− group, *n* = 13), or those who underwent CT and had a perfusion defect (CT and defect + group, *n* = 27), respectively (Table 1). There were no significant differences in history and clinical presentation among the three groups. Compared to patients with non-CT, the CT and defect+ patients were significantly younger (64 ± 17 vs. 71 ± 11 years, *P* = 0.020) and had a higher prevalence of culprit lesions of the left circumflex [13 (48.2%) vs. 32 (20.5%), *P* = 0.006]. The CT and defect+ also had higher contrast medium volume in the artery and vein compared to non-CT [261 (214, 290) vs. 140 (120, 175) mL, *P *< 0.001]. There was no significant difference in the incidence of CIN among the three groups.

**Table 1:** Patient characteristics.

Variables	Total *N* = 196	Not CT group *N* = 156	CT and defect− group *N* = 13	CT and defect+ group *N* = 27	*P* value
Age (%), years	71 ± 13	71 ± 11	73 ± 14	64 ± 17*	0.021
Female sex, *n* (%)	44 (22.4)	36 (23.1)	5 (38.4)	3 (11.1)	0.13
Body mass index, mean ± SD, kg/m^2^	24.0 ± 3.9	23.8 ± 3.7	23.9 ± 5.0	24.6 ± 4.7	0.66
Past history					
Hypertension, *n* (%)	162 (82.7)	128 (82.1)	12 (92.3)	22 (81.5)	0.63
Diabetes, *n* (%)	89 (45.4)	73 (46.8)	4 (30.8)	12 (44.4)	0.53
Dyslipidemia, *n* (%)	141 (71.9)	111 (71.2)	10 (76.9)	20 (74.1)	0.87
Smoking, *n* (%)	116 (59.5)	96 (61.5)	5 (41.7)	15 (55.6)	0.36
History of hemodialysis	24 (12.2)	23 (14.7)	1 (7.7)	0	0.09
History of PCI, *n* (%)	69 (35.2)	59 (37.8)	3 (23.1)	7 (25.9)	0.31
History of CABG, *n* (%)	12 (6.1)	12 (7.7)	0	0	0.19
Clinical presentation					
Systolic blood pressure ± SD, mmHg	146 ± 28	145 ± 28	160 ± 33	149 ± 29	0.17
Heart rate ± SD, /min	84 ± 19	84 ± 18	85 ± 22	83 ± 21	0.92
Killip classification ≥2, *n* (%)	44 (22.4)	35 (22.4)	3 (23.1)	6 (22.2)	1.00
Tracheal intubation, *n* (%)	9 (4.6)	7 (4.5)	0	2 (7.4)	0.57
Medications					
β blocker, *n* (%)	53 (27.0)	42 (26.9)	3 (23.1)	8 (29.6)	0.91
Renin-angiotensin system inhibitors, *n* (%)	89 (45.4)	76 (48.7)	4 (30.8)	9 (33.3)	0.18
Calcium channel blocker, *n* (%)	76 (38.8)	65 (41.7)	3 (23.1)	8 (29.6)	0.24
Statin, *n* (%)	85 (43.6)	73 (46.8)	4 (30.8)	8 (29.6)	0.16
Laboratory data					
Hemoglobin, mean ± SD, g/dL	13.3 ± 2.2	12.9 ± 2.2	13.3 ± 1.9	13.8 ± 2.3	0.15
Albumin, mean ± SD, g/dL	3.8 ± 0.6	3.8 ± 0.7	4.1 ± 0.4	4.0 ±0.4	0.13
Creatinine at baseline, median (IQR), mg/dL	0.88 (0.71, 1.19)	0.94 (0.75, 1.33)	0.73 (0.58, 0.99)	0.75 (0.69, 0.92)*	0.010
Creatinine ≥1.5 mg/dL, *n* (%)	37 (18.9%)	34 (21.8%)	1 (7.7%)	2 (7.4%)	0.12
HbA1c, median (IQR), %	6.1 (5.7, 6.8)	6.2 (5.8, 6.8)	6.0 (5.5, 7.1)	6.0 (5.6, 6.7)	0.36
CK at baseline, median (IQR), IU/L	129 (75, 288)	130 (77, 280)	109 (66, 145)	130 (74, 344)	0.39
Peak CK, median (IQR), IU/L	233 (99, 675)	218 (95, 742)	109 (66, 152)	416 (187, 652)	0.013
NT-proBNP, median (IQR), pg/mL	535 (217, 2657)	612 (237, 3863)	487 (145, 868)	345 (154, 1140)	0.22
NT-proBNP ≥900 pg/mL, *n* (%)	56 (28.6)	47 (30.1)	1 (7.7)	8 (29.6)	0.22
Classification of acute coronary syndrome					
NSTEMI CK+, *n* (%)	97 (49.5)	72 (46.2)	3 (23.1)	22 (81.5)*	< 0.001
NSTEMI CK−, *n* (%)	69 (35.2)	58 (37.2)	6 (46.2)	5 (18.5)	0.12
NSTE-ACS UA, *n* (%)	30 (15.3)	26 (16.7)	4 (30.8)	0*	0.024
Culprit vessel of ACS					
Left main trunk, *n* (%)	10 (5.1)	8 (5.1)	1 (7.7)	1 (3.7)	0.87
LAD, *n* (%)	89 (45.4)	75 (48.1)	4 (30.8)	10 (37.0)	0.31
LCX, *n* (%)	50 (25.5)	32 (20.5)	5 (38.5)	13 (48.2)*	0.005
RCA, *n* (%)	45 (23.0)	39 (25.0)	3 (23.1)	3 (11.1)	0.29
Bypass, *n* (%)	2 (1.0)	2 (1.3)	0	0	0.77
Single vessel disease, *n* (%)	75 (39.7)	60 (38.5)	4 (30.8)	11 (40.7)	0.83
Contrast medium volume in artery, median (IQR), mL	140 (120, 175)	140 (120, 175)	140 (123, 162)	150 (120, 180)	0.67
Contrast medium volume in artery and vein, median (IQR), mL	153 (125, 213)	140 (120, 175)	234 (204, 255)	261 (214, 290)*	< 0.001
Incidence of CIN, *n* (%)	21 (12.2)	17 (12.8)	1 (8.3)	3 (11.1)	0.89
Time of arrival to CAG, median (IQR), min	181 (70, 941)	202 (72, 980)	351 (48, 1325)	93 (59, 175)^*^	0.007
CAG within 2 hours of arrival, *n* (%)	76 (38.8)	53 (34.0)	6 (46.2)	17 (63.0)*	0.015

Data are mean ± SD, median (interquartile range), or *n* (%). Final column reflects overall group differences.

* *p* < 0.05 vs. Not CT; CABG, Coronary artery bypass grafting; CAG, Coronary angiography; CK, Creatine kinase; CIN, Contrast-induced nephropathy; CT, Computed tomography; LAD, Left anterior descending artery; LCX, Left circumflex artery; NT-proBNP, N-terminal pro-B-type natriuretic peptide; NSTEMI CK+, Non-ST segment elevation myocardial infarction with elevated CK; NSTEMI CK−, Non-ST segment elevation myocardial infarction with elevated cTnI only; NSTE-ACS UA, Non-ST-elevation acute coronary syndrome without elevated cTnI; PCI, Percutaneous coronary intervention; RCA, Right coronary artery.

### The time from arrival at the hospital to CAG

**Figure 2:**
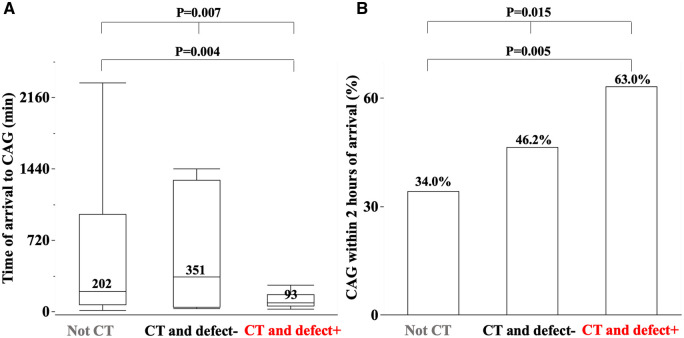
The median time of arrival to coronary angiography (CAG) (**A**) and percentage of CAG within 2 h of arrival among three groups (**B**). The detail is shown in the text.

Figures 2A,B show the time from arrival to CAG [non-CT 202 (72, 980), CT and defect− 351 (48, 1,325), CT and defect+ 93 (59, 175) min, *P* = 0.015] and the percentage of CAG within 2 h of arrival [non-CT 53 (34.0%), CT and defect− 6 (46.2%), CT and defect+ 17 (63.0%) patients, *P* = 0.007], respectively. Compared to non-CT, patients with CT and defect+ patients had a shorter time from arrival to CAG and a higher percentage of CAG within 2 h of arrival.

In the univariate analysis, non-gated CT was a predictor of CAG within 2 h of arrival (OR 2.63, 95% CI 1.29–5.34, *P* = 0.007) (Table 2). In the multivariate analysis, non-gated CT was a predictor of CAG within 2 h of arrival (OR 2.34, 95% CI 1.12–4.89, *P* = 0.024). Age, the procedure of tracheal intubation, NT-proBNP ≥900 pg/mL, creatinine ≥1.5 mg/dL, and NSTEMI were not significant factors (Table 2).

**Table 2:** Predictors of coronary angiography (CAG) within 2 h of arrival.

	Univariate		Multivariate	
	Odds ratio (95% CI)	*P*-value	Odds ratio (95% CI)	*P*-value
Age	0.99 (0.96–1.01)	0.22	0.99 (0.96–1.01)	0.41
Female sex	0.88 (0.44–1.76)	0.71		
Diabetes	0.96 (0.54–1.70)	0.88	0.90 (0.48–1.66)	0.71
Killip classification ≥2, *n* (%)	1.61 (0.82–3.17)	0.17		
Tracheal intubation, *n* (%)	0.44 (0.09–2.16)	0.31	0.42 (0.08–2.30)	0.29
Creatinine ≥1.5 mg/dL	0.52 (0.80–1.06)	0.11	0.62 (0.27–1.45)	0.26
NT-proBNP ≥900 pg/mL, *n* (%)	0.93 (0.49–1.76)	0.82	1.06 (0.52–2.17)	0.86
Single vessel disease, *n* (%)	1.21 (0.67–2.19)	0.53		
NSTEMI	2.34 (0.95–5.75)	0.06	2.28 (0.91–5.72)	0.08
Elevated CK at baseline	1.09 (0.58–2.06)	0.78		
Non-gated CT	2.63 (1.29–5.34)	0.007	2.34 (1.12–4.89)	0.024

CK, creatine kinase; CT, computed tomography; NSTEMI, non-ST-segment elevation myocardial infarction; NT-proBNP, N-terminal pro-B-type natriuretic peptide.

### Initial and peak CK

**Figure 3:**
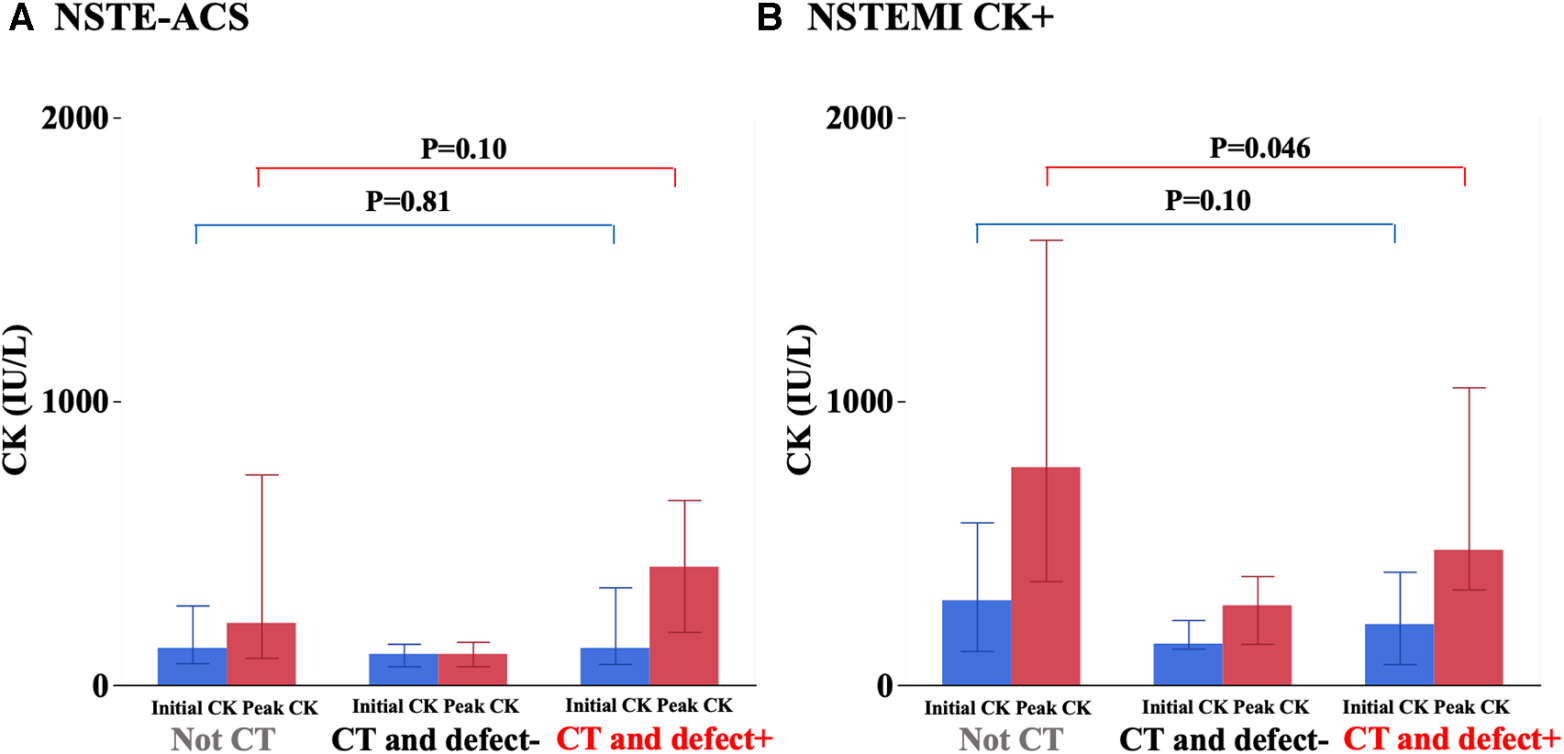
Initial and peak creatine kinase (CK) levels among three groups. The detail is shown in the text.

Initial and peak CK were not statistically different among the three groups (Table 1). There was no significant difference in peak CK between the non-CT group and the CT and defect+ group (Figure 3A). As only the CT and defect+ group had a higher prevalence of NSTEMI CK+, we also analyzed only NSTEMI CK+. There was no significant difference in the initial CK levels among the three groups of NSTEMI CK+ only [non-CT 298 (120, 573), CT and defect− 145 (128, 229), CT and defect+ 214 (73, 399) IU/L, *P* = 0.19]. On the other hand, there was a difference in peak CK levels among the three groups [non-CT 767 (366, 1,569), CT and defect− 280 (145, 384), CT and defect− 475 (337, 1,049) IU/L, *P* = 0.015]. Peak CK levels in the CT and defect+ patients were significantly lower compared to the non-CT group (Figure 3B).

### Perfusion defects on CT

**Figure 4:**
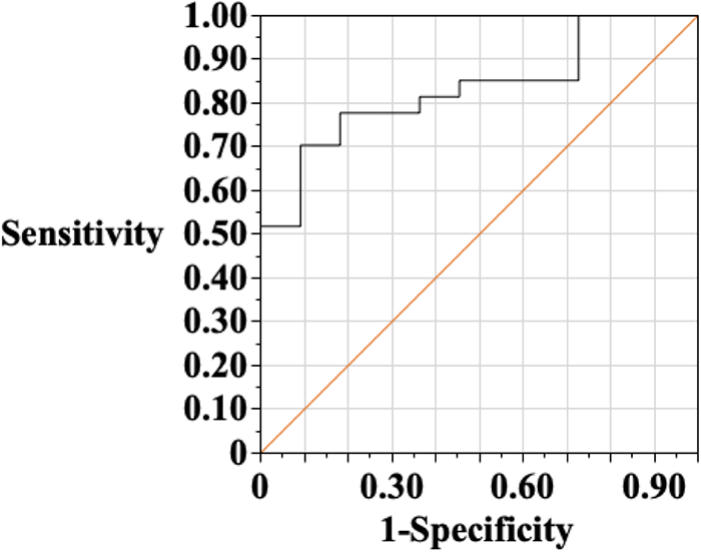
Receiver-operating characteristic (ROC) analysis of peak creatine kinase (CK) in the finding of perfusion defect on CT. The area under the curve was 0.83, and the best discriminate value of the peak CK was 301 IU/L. Then, the sensitivity is 70.4%, and specificity of 91.8%. When NSTE-ACS with a peak CK value of over 416 IU/L was selected as the cutoff value, the false-positive rate in the determination of abnormal perfusion by CT was 0%.

Figure 4 shows the results of ROC analysis of peak CK levels in the finding of the CT-derived perfusion defect. The area under the curve was 0.83, and the best discriminating value of the peak CK was 301 IU/L. NSTE-ACS with peak CK levels over 416 IU/L had a 0% false positive rate for determining the CT-based perfusion defect.

**Figure 5:**
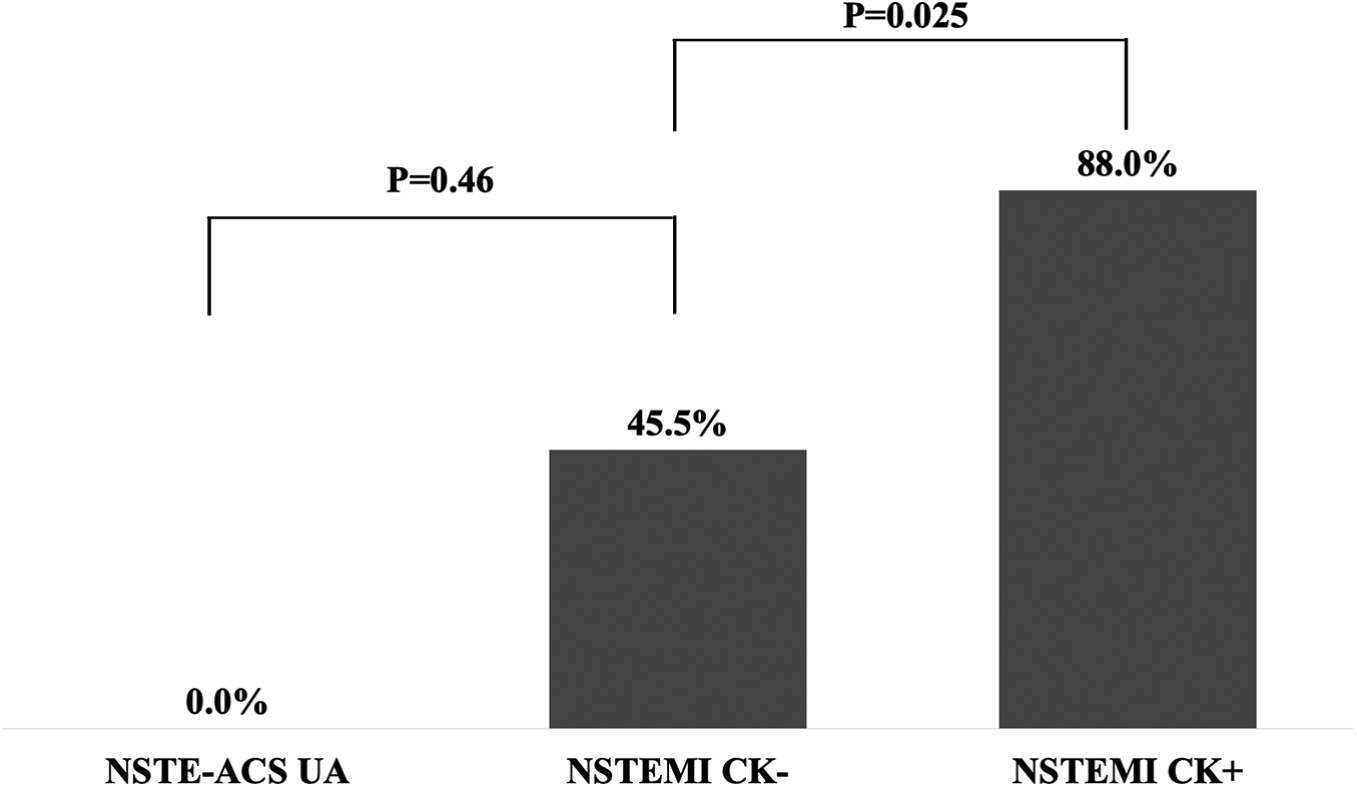
The percentage of perfusion defect on CT on each type of NSTE-ACS.

The non-gated CT characteristics are shown in Figure 5. The sites of perfusion defect on CT were all correlated with the responsible lesions on CAG. In 88.0% of the NSTEMI CK+ patients, perfusion defects were identified on CT images, while those were identified in 45.5% of the NSTEMI CK− patients. None of the NSTE-ACS UA patients had CT-based perfusion defects.

### Factors associated with performing CT

We investigated the factors for performing CT in Table 3. Patients with LCX as the culprit lesion were significantly more likely to perform CT in both univariate (OR 3.17, 95% CI 1.52–6.60, *P* = 0.002) and multivariate analysis (OR 3.09, 95% CI 1.46–6.57, *P* = 0.003), but unstable hemodynamic conditions such as low systolic blood pressure values, Killip classification ≥2, and NT-proBNP ≥900 pg/mL were not associated with performing CT.

**Table 3:** Predictors of performing CT.

	Univariate		Multivariate	
	Odds ratio (95% CI)	*P*-value	Odds ratio (95% CI)	*P*-value
Age	0.97 (0.95–1.00)	0.06	0.98 (0.94–1.01)	0.11
Systolic blood pressure	1.00 (1.00–1.02)	0.12		
Heart rate	1.00 (0.98–1.02)	0.82		
Killip classification ≥2	1.00 (0.44–2.30)	0.99		
NT-proBNP ≥900 pg/mL	0.70 (0.31–1.58)	0.39	0.96 (0.39–2.36)	0.92
Elevated CK at baseline	0.96 (0.44–2.11)	0.93		
Creatinine ≥1.5 mg/dL	0.29 (0.08–1.00)	0.05	0.32 (1.13–5.00)	0.08
LAD	0.58 (0.28–1.20)	0.14		
LCX	3.17 (1.52–6.60)	0.002	3.09 (1.46–6.57)	0.003
RCA	0.53 (0.21–1.36)	0.16		

CK, creatine kinase; CT, computed tomography; LAD, left anterior descending artery; LCX, left circumflex artery; NT-proBNP, N-terminal pro-B-type natriuretic peptide; RCA, right coronary artery.

## Discussion

The present study is the first to report on the clinical impact of non-gated CT in patients with NSTE-ACS. There were three major findings. First, the CT and defect+ group had a shorter time from arrival to CAG compared to the non-CT group. Second, in patients with NSTEMI CK+, the CT and defect+ group had a lower peak CK than that in the non-CT group. Third, the present study revealed a close association between the CT-based perfusion defect and the type of NSTE-ACS or peak CK levels.

In comparison with previous studies, patient characteristics in this study, such as age, prevalence of female and coronary risk factors, and creatinine levels, were similar to those in the J-MINUTE study NSTEMI group (12), which is a prospective observational multicenter study about the Japanese registry of AMI. On the other hand, peak CK levels of our study patients were lower than J-MINUTE study [649 (354, 1,340) IU/L vs. 1,151 (702, 2,076) IU/L in NSTEMI CK + patients, 107 (70, 171) IU/L vs. 161 (101, 254) IU/L in NSTEMI CK− patients]. Thus, myocardial injury in the patients in this study may have been relatively mild. Previous studies have shown the effectiveness of CCTA in patients with suspicion of ACS (13–15). A previous randomized controlled study showed that CCTA has a high diagnostic accuracy to rule out clinically significant coronary artery disease in 1,023 patients with NSTE-ACS (16). The current recommendation (2023) of the European Cardiology Guide specifies that CCTA is indicated in patients without very high- or high-risk features and a low index of suspicion for NSTE-ACS (17). Cautiously, routine early CCTA is not recommended in patients with suspected ACS (III B) (17). The CCTA can be also useful in situations when CAG is hesitant due to the risk of complications (18). Nonetheless, it is clinically difficult to perform CCTA to explore cardiac diseases in all patients because of the following situations. ACS patients are often unstable because of their symptoms, so patients do not always have breath-holds, stable heart rates, or the ability to follow an order. Some patients cannot tolerate nitro drugs for coronary vasodilation or beta-blockers for heart rate reduction because of arrhythmias or blood pressure. Moreover, ACS patients are sometimes initially suspected of having respiratory or gastrointestinal diseases. The CCTA also has a practical issue in that its 24-hour service is not always available (17). On the other hand, a non-gated CT scan does not require any examination procedures or image construction, allowing for rapid image interpretation. Importantly, the performance of non-gated enhanced CT followed by invasive CAG (with or without percutaneous or surgical revascularization) in NSTE-ACS patients is controversial.

Our results in this study support that non-gated CT detects NSTE-ACS with elevated CK levels, and thus, it provides a decision criterion for early revascularization.

The CT and defect+ groups had a shorter time from arrival to CAG. This result is clinically plausible because the CT-derived perfusion defects suggesting the exposition of severe ischemia or infarction in the non-small extent myocardium indicates the need for immediate revascularization. This finding is valuable especially in uncertain situations whether the urgent CAG is required or not due to a lack of typical ECG changes or symptoms of ACS. Particularly, non-gated CT can be more useful in detecting acute myocardial infarction caused by circumflex or peripheral lesions that present limited electrocardiographic changes. Patients with responsible lesions in the circumflex lesions were more likely to have non-gated CT in this study. Moreover, non-gated CT was significantly associated with CAG within 2 h; especially, among the patients with NSTEMI CK+, peak CK levels were lower in the CT and defect+ group than those in the non-CT group [475 (337, 1,049) vs. 767 (366, 1,569) IU/L, *P* = 0.046]. These results might be explained by the fact that we could have predicted the exposition to severe ischemia or infarction by the CT-based perfusion defects, even in cases with an unknown or not elevated myocardial deviation enzyme on arrival. However, significant differences were found in the time of arrival to CAG and peak CK in this study as mentioned above, but the sample size was small and the confidence interval was wide. In NSTEMI CK+ patients, baseline CK was not completely matched between the non-CT and CT and defect+. At least, this study showed that non-gated CT is effective for early diagnosis and treatment without significantly increasing the time to CAG or peak CK. Importantly, CT was performed at the decision of clinicians, but our physicians have indications for CT for the following reasons: (1) the cause of chest pain may be ACS but was not positively considered, and (2) the patient had a possibility of myocardial infarction due to aortic dissection. We also hesitated to perform CT scans for the following reasons: (1) patients with severe renal failure; (2) patients who had severe heart failure (NYHA Class IV) and were not expected to tolerate the preload caused by contrast medium; (3) older patients who had difficulty holding their breath for CT imaging; (4) obvious diagnosis of ACS. As a result, patients with higher age, serum creatinine level, and NT-proBNP ≥900 pg/mL were less likely to undergo CT, although these differences were not statistically significant (Table 3).

Our results demonstrated a close association between the type of NSTE-ACS and non-gated CT. The sensitivity of CT to detect acute myocardial infarction with elevated CK was good in this study, as in previous reports (19). Even among the NSTEMI CK− patients, 45.5% of these patients had perfusion defects. This result has not been previously reported, although the number of patients is limited.

## Clinical implication

Identifying and appropriately treating higher-risk patients with NSTE-ACS is clinically crucial because they derive the most benefit from a more invasive approach earlier in their course (2, 3). High-risk signs include ongoing angina, arrhythmias, dynamic ECG changes, and hemodynamic compromise. Elevated cTnI is also a sign of early invasive intervention. The randomized study (RIDDLE-NSTEMI study) revealed that an immediate invasive strategy (within 2 h of admission) in NSTE-ACS patients with elevated cTnI is associated with lower rates of death or new MI compared with the delayed invasive strategy (within 72 h of admission) at early and midterm follow-up. However, cTnI is often not elevated in the hyperacute phase of myocardial infarction. Therefore, patients with suspected myocardial infarction are retested for myocardial deviation enzymes a few hours later. This study showed that the CT-based perfusion defect could be a pivotal indicator for early revascularization in patients with NSTE-ACS.

It is well known that the peak serum CK levels after acute MI, as a reflection of infarct size, are strongly associated with subsequent mortality (20, 21). Also, in patients with NSTE-ACS, elevated CK is a factor associated with the occurrence of events such as all-cause death, non-fatal MI, non-fatal stroke, heart failure, and urgent revascularization up to 30 days (12). Therefore, efforts to minimize peak CK levels are warranted in NSTE-ACS patients. It is also important to understand the predictors of elevated CK levels in the clinical practice of acute myocardial infarction. This study showed that the CT-based perfusion defect is a robust predictor for elevation CK levels. In the present study, all patients with NSTE-ACS with peak CK >416 IU/L had perfusion defects. There have been limited data on the association between peak CK levels and perfusion defect on non-gated CT. In a non-gated CT study, the maximum peak CK in cases without perfusion defect was 470 IU/L, which is consistent with our results (19). Taken together, CK is not expected to be markedly elevated in patients with NSTE-ACS who had no perfusion on non-gated CT.

## Limitations

Our study has several limitations. First, it was a single-center study with a relatively small sample size. In particular, the sample size undergoing CT was small. Furthermore, non-gated CT was relatively performed for NSTE-ACS with LCX as the culprit lesion and less for NSTE-ACS with the left anterior descending artery (LAD) or right coronary artery (RCA). Although this study could indicate that LCX is effective for early diagnosis of NSTE-ACS in responsible lesions, the same may not be suggested for LAD and RCA. However, this study also suggested that non-gated CT has the potential to provide additional information for NSTE-ACS of culprit lesions with LAD and RCA, which are difficult to diagnose from symptoms and ECG, without increasing CIN and the time to CAG. Second, study patients are a group judged as relatively critical by the clinicians. In fact, 170 (86.7%) patients in this study were selected for early invasive therapy, i.e., within 24 h from hospital arrival. Some NSTE-ACS patients who did not have typical symptoms, an elevated cTnI at arrival, or perfusion defect might not have visited emergency rooms. Therefore, it cannot be considered this study deals with all cases of NSTE-ACS. Third, the study design was retrospective and single-center. Clinicians at our institution realize the effectiveness of non-gated CT in the practice of NSTE-ACS and are well-trained in the detection of perfusion defects (6, 7). Fourth, the authors assessed the perfusion defect visually rather than by CT attenuation thresholds. Accurate measurement of CT attenuation may be difficult on non-gated CT because of motion or beam hardening artifacts, and thus, visual assessment seems to be a more clinically suitable way to assess the myocardium. Finally, some patients scheduled for urgent CAG might have non-gated CT only to check aortic dissection. Physicians who did not check perfusion defects performed urgent CAG regardless of perfusion defect or not. In such a scenario, the presence or absence of perfusion defect might not have affected the timing of CAG.

## Conclusions

Our study suggests that non-gated CT is valuable for early diagnosis and treatment strategy of NSTE-ACS. This study also showed that perfusion defects on CT were indicative of peak CK levels.

## Data Availability

The original contributions presented in the study are included in the article/Supplementary Material, and further inquiries can be directed to the corresponding author.
